# Single‐Cell Insights Into Cellular Response in Abdominal Aortic Occlusion‐Induced Hippocampal Injury

**DOI:** 10.1111/cns.70154

**Published:** 2025-01-20

**Authors:** Changhong Ren, Ling Kui, Jun Xu, Fang Tong, Xiaojie Wang, Jianping Ma, Xiaomei Tian, Guoyun Wang, Feng‐Yong Liu, Sijie Li, Xunming Ji

**Affiliations:** ^1^ Beijing Key Laboratory of Hypoxia Translational Medicine, Xuanwu Hospital, Center of Stroke, Beijing Institute of Brain Disorder Capital Medical University Beijing China; ^2^ Bioinformatics Center Shenzhen Qianhai Shekou Free Trade Zone Hospital Shenzhen China; ^3^ Department of Neurology Shenzhen Qianhai Shekou Free Trade Zone Hospital Shenzhen China; ^4^ Department of Interventional Radiology, Senior Department of Oncology Fifth Medical Center of PLA General Hospital Beijing China

**Keywords:** abdominal aortic occlusion, astrocyte, hippocampus, Ischemia‐reperfusion, OPC, scRNA‐seq

## Abstract

**Objective:**

Ischemia–reperfusion of the abdominal aorta often results in damage to distant organs, such as the heart and brain. This cellular heterogeneity within affected tissues complicates the roles of specific cell subsets in abdominal aorta occlusion model (AAO) injury. However, cell type–specific molecular pathology in the hippocampus after ischemia is poorly understood.

**Aims:**

In this study, we adopted a mouse AAO to investigate the single‐cell transcriptome in the hippocampi in AAO mice.

**Methods:**

Male C57BL/6 mice (8 weeks old) were used to create an AAO model, with animals divided into Sham and I/R groups. The I/R group was subjected to 2 h of ischemia followed by 24 h of reperfusion, after which hippocampal tissues were collected for single‐cell RNA sequencing and histological analysis. Behavioral tests, including the Rotarod, Y‐maze, and new object recognition tests, were performed daily for 28 days post‐surgery to evaluate neurological function. A total of 62,624 cells were corresponding 7 cell types with neuronal, glial, and vascular lineages. We next analyzed cell‐specific gene alterations in AAO mice and the function of these cell‐specific Genes.

**Results:**

AAO injury upregulated astrocyte and oligodendrocyte precursor cell (OPC) proportions (*p*‐value < 0.05). Astrocytes showed unique gene expression related to neurogenesis and mRNA processing. Five distinct astrocyte subtypes emerged post‐injury. OPCs exhibited enhanced synapse organization. Microglia activation and the elevated expression level of the epithelial cell oxidative phosphorylation protein–protein interaction (PPI) module indicate an inflammatory response and metabolic changes in response to AAO injury.

**Conclusions:**

Our scRNA‐seq analysis provides insights into transcriptional changes at the single‐cell level in response to AAO‐induced hippocampal injury. This study illustrates how the hippocampal region responds to such injury and identifies potential therapeutic targets for intervention, thereby paving the way for future research and treatment strategies.

## Graphical abstract

In this study, single‐cell RNA sequencing (scRNA‐seq) was used to analyze the cellular response in the hippocampus of a mouse model with abdominal aortic occlusion (AAO) injury. We identified changes in cell proportion, including astrocytes and oligodendrocyte precursor cells (OPCs), and observed gene expression changes associated with neurogenesis and inflammation.

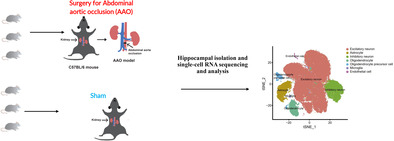

**Abbreviations**
AAO: abdominal aortic occlusion
CNS: central nervous system
DEG: different expressed gene
I/R: ischemia/reperfusion
OPC: oligodendrocytes precursor cell
PPI: protein–protein interaction
scRNA‐seq: single‐cell RNA sequencing
t‐SNE: t‐distributed stochastic neighbor embedding
UMAP: uniform manifold approximation and projection

## 1 Background

Abdominal aortic ischemia/reperfusion (AAO) induced ischemia/reperfusion (I/R) injury arises in diverse clinical contexts, such as perivascular surgery, repair of abdominal aortic aneurysms or dissection I/Rs, acute abdominal aortic thromboembolism, emergency trauma surgery [1, 2], and balloon occlusion therapy following abdominal blood loss [3]. This condition is particularly relevant in high‐risk scenarios like battlefield injuries and severe trauma, where rapid exsanguination due to non‐compressible torso hemorrhage significantly increases mortality [4]. Although Resuscitative Endovascular Balloon Occlusion of the Aorta (REBOA) is a vital technique in trauma care to control hemorrhage and potentially reduce mortality rates [4], its use can also precipitate AAO‐induced I/R injury [5]. This dual role highlights the complexity of managing such injuries and the need for an in‐depth understanding of their broad biological impacts, particularly on vital organs.

The systemic effects of AAO I/R injury extend beyond the abdominal aorta and its proximal organs, severely impacting distant organs like the heart, lungs, and notably the brain [6, 7, 8]. The hippocampus, a brain region with high metabolic demand and a critical role in cognitive functions, is particularly vulnerable [9]. This susceptibility underscores the importance of exploring the molecular and cellular responses of the hippocampus to AAO‐induced I/R injury [8]. Our previous study demonstrated that AAO‐induced I/R leads to injury in the pyramidal cells of the CA1 region of the hippocampus [6]. Understanding this damage could lead to better management strategies for minimizing harm and enhancing recovery in affected patients.

The hippocampus, with its intricate structure and diverse cellular composition, poses significant challenges in understanding the molecular mechanisms of AAO‐induced I/R injury. The regulation of gene expression within these cells is crucial for mediating biological responses such as cell death, inflammation, and tissue recovery. Notably, various cell types within the central nervous system (CNS), including those in the hippocampus, exhibit distinct sensitivities to ischemia. Previous research has shown that different cell types in the central nervous system respond differently to hypoxic conditions [10, 11]. Cultured neurons and glial cells exhibit differences in gene expression responses under hypoxic conditions [12]. Despite this, the specific responses of different CNS cell types to AAO‐induced I/R have not been thoroughly examined in vivo on a genome‐wide scale. This variability in cellular response underscores the importance of employing advanced techniques like single‐cell RNA sequencing (scRNA‐seq) to precisely elucidate the cellular and molecular dynamics specific to each cell type [13, 14, 15, 16].

To address these challenges, we have utilized scRNA‐seq technologies, leveraging the advanced 10x Genomics platform. This approach allows us to dissect the transcriptional landscape of thousands of individual cells from the hippocampus of mice subjected to AAO. Such detailed analysis is crucial, as the injury and the subsequent biological responses unfold rapidly and have a narrow window for effective therapeutic intervention. By focusing on the acute phase of AAO‐induced I/R injury, our study aimed to provide a comprehensive understanding of the cellular dynamics and molecular mechanisms that dictate the outcome of this complex condition.

## 2 Methods

### 2.1 Animals

Eight‐week‐old male C57BL/6 mice were used in all experiments. All animals were purchased from SPF (Beijing) Biotechnology Co. Ltd. and housed separately in a room with a 12‐h light–dark cycle. In addition, the room has a controllable temperature (22°C ± 2°C) and provides sufficient food and water for animals. All animal experiments in this study were approved and reviewed by the Institutional Animal Care and Use Committee of Capital Medical University on October 14, 2021 (approval number: 2021/255) and complied with the Guide for the Care and Use of Laboratory Animals.

### 2.2 AAO Model and Behavioral Assessment

**FIGURE 1:**
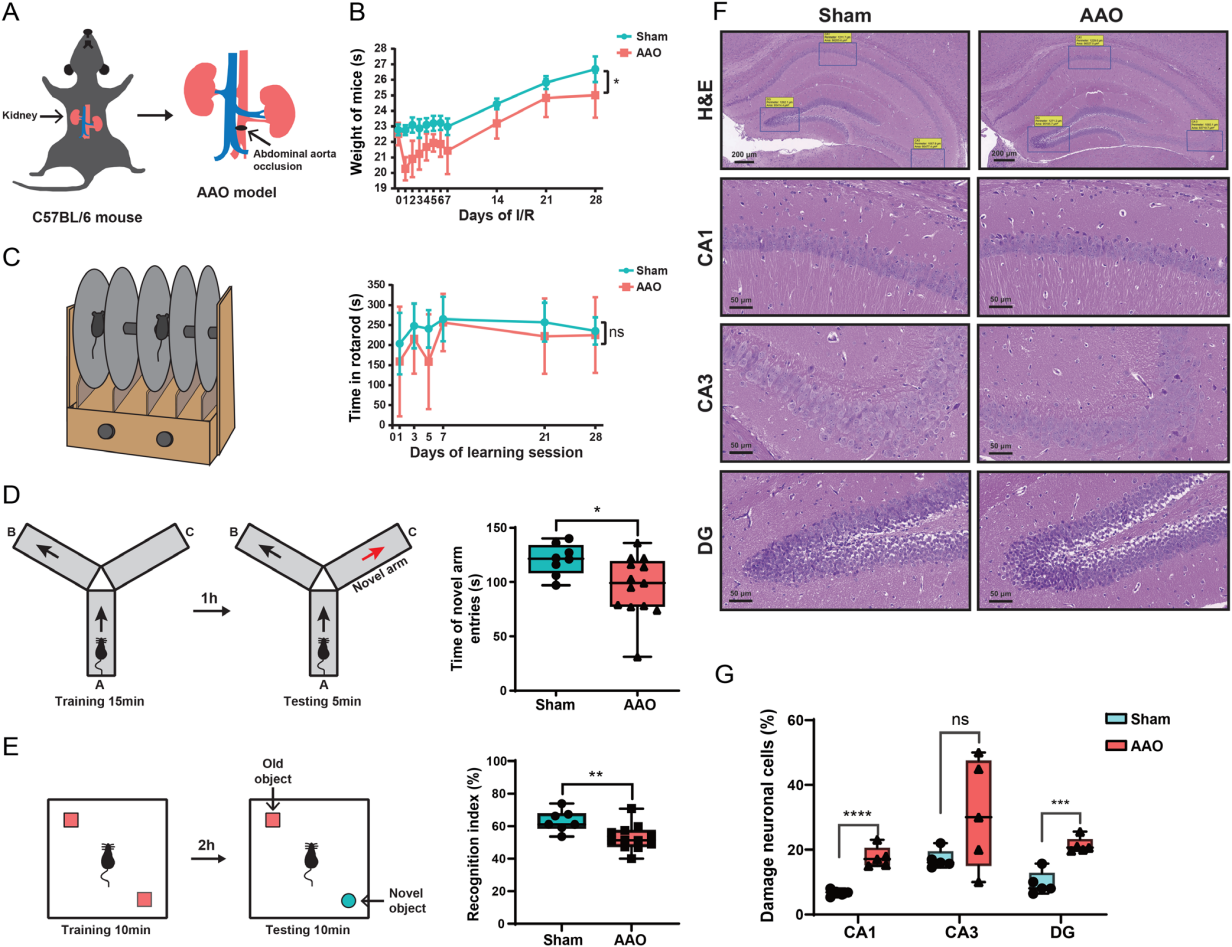
AAO induced neurological function impairment and pathological damage in Mice. (A) Schematic, presentation illustrating the AAO in C57BL/6 mice. (B) Weight curve of mice after AAO model for 28 days. (C) Schematic, presentation images and quantitative graph of rotarod (RR) test. The time of staying in RR revealed the impact of AAO on motor coordination and balance in mice. (D) Schematic, presentation images and quantitative graph of Y‐maze assay. The time of entering the novel arm indicated the impact of AAO on spatial recognition in mice. (E) Schematic, presentation images and quantitative graph of new object recognition (NOR) test. The recognition index illustrated the effect of AAO on recognition memory function in mice. (F) Representative images of H&E staining of brain, the deep staining of CA1, CA3, and DG represented the damage neuronal cells in the hippocampus. The scale bar for the entire hippocampus region is 200 μm and for the local region (CA1, CA3, and DG) is 20 μm. (G) The graph represents quantitative analysis of the damage neuronal cells in the hippocampus. Data are presented as mean ± SEM, *n* is 5–12 per group vs. Sham group, *p < 0.05, **p < 0.01. ***p < 0.001, ****p < 0.0001.

In this study, the AAO model was employed to examine I/R injury in the brain as previous descript [6, 17]. Mice were randomly divided into two groups: Sham group and I/R groups with ischemia durations of 2 h. Anesthesia was induced using a mixture of 1.5% enflurane, 30% O_2_, and 68.5% N_2_O. After shaving and disinfecting the abdomen, a midline incision was made. The Sham group underwent a laparotomy only. In the I/R groups, the abdominal aorta, located 0.5 cm below the left renal artery, was occluded with sutures for 2 h, followed by varying reperfusion periods as experiment required (Figure 1A).

### 2.3 Neurological Function Test

All animals were habituated to the new environment for at least 2 days before behavioral test. All of the behavioral test were conducted in a blinded manner with at least a 2‐h interval between different assays. The order of tests is ratarod, Y‐maze, and new object recognition.

### 2.4 Rotarod (RR) Test

The rotarod (RR) test evaluated the impact of the AAO model on motor coordination and balance in mice. Mice were placed on a horizontally rotating rod and required to walk to maintain balance. The time taken to fall off was recorded to assess motor function. Mice were acclimatized to the device for 5 min at rest. Over the first 3 days, they were trained at 4 rpm with three 5‐min trials per day and 60‐min intervals. Mice that remained on the rod for at least 60 s were selected for further studies. Latency to fall off the rod at 5 rpm for 5 min was recorded on 1, 3, 5, 7, 21, and 28 days post‐AAO.

### 2.5 Y‐Maze Test

The Y‐maze test assessed the impact of the AAO model on mice's spatial recognition memory. This test was conducted on the 28th day post‐AAO. The Y‐maze has three identical arms (26 cm × 4 cm) at 120° angles. During training, mice explored arms A and B for 15 min, with arm C blocked. After 1 h, arm C was opened for the test phase, and mice explored all three arms for 5 min. The time taken to enter the novel arm C was recorded.

### 2.6 New Object Recognition (NOR) Test

The New Object Recognition (NOR) test evaluated the impact of the AAO model on mice's recognition memory. This test was conducted on the 28th day post‐AAO. Mice were tested in a 25 × 25 cm black arena, 40 cm high. Initially, two identical objects were presented for familiarization. Mice explored these objects for 10 min. Two hours later, one object was replaced with a novel one, and mice explored for another 10 min (Figure 1E). The frequency, time, and distance of exploration were recorded. The recognition index (RI) was calculated as RI = (novel object/(novel object + old object)) × 100%.

### 2.7 Hematoxylin and Eosin (H&E) Staining Analysis

The mice were anesthetized and transcranial perfused with cold normal saline 28 days post‐AAO, and then, brain tissues were taken and fixed with 4% paraformaldehyde fixative solution, embedded in paraffin, and sectioned to an 4.5 μm thickness slices. Subsequently, hematoxylin–eosin (H&E) staining was used to observe the morphological changes of hippocampal tissues, as described in previous study [6]. Briefly, all slices were scanned by an automatic microscopic scanner (Nikon, Japan). Five fields were randomly selected from slices in different groups. The nuclei is appear blue‐purple, while the cytoplasm and extracellular matrix are pink. The neurohistological analysis involves counting and quantifying the damage neuronal cells in hippocampus at high‐power fields of sections.

### 2.8 Tissue Dissociation and Nuclei Isolation

After 2 h of AAO treatment and 24 h of reperfusion in mice, cells were isolated in preparation for scRNA‐seq. After the mice had been exsanguinated by transcranial physiological saline (PS, Thermo Fisher Scientific) perfusion, hippocampus tissue was isolated from Hippocampus and washed by 1× phosphate buffered saline (PBS, Thermo Fisher Scientific), then quickly frozen and stored in liquid nitrogen. Nuclei extraction was separated by mechanical extraction method. Firstly, put the tissues into 2 mL Dounce homogenizer set and thawed in homogenization buffer (containing 20 mM Tris pH 8.0, 500 mM sucrose, 0.1% NP‐40, 0.2 U/μL RNase inhibitor, 1× protease inhibitor cocktail, 1% bovine serum albumin) (BSA, Thermo Fisher Scientific), and 0.1 mM DTT. Use Dounce pestle A to grind the tissue 10 times, filter with 70‐μm cell filter, and then grind with Dounce pestle B 10 times, filter with 30‐μm cell filter. Centrifuge at 500 *g* for 5 min at 4°C to pellet the nuclei, and resuspend in the blocking buffer containing 1% BSA and 0.2 U/μL RNase inhibitor in 1× PBS. Centrifuge again at 500 *g* for 5 min and resuspend with Cell Resuspension Buffer (MGI).

### 2.9 Single‐Cell RNA Library Construction and Sequencing

The mRNA capture was operated on DNBelab C4 device (MGI). Complete cDNA amplification and library construction according to the MGI DNBelab C series reagent Kit (MGI, 940‐000047‐00) [18]. In brief, the single‐cell suspensions were converted to barcoded libraries through steps including droplet encapsulation, emulsion breakage, mRNA captured bead collection, reverse transcription, cDNA amplification, and purification. cDNA production was sheared to 250–400 bp, and indexed sequencing libraries were constructed according to the manufacturer's protocol. Qualification was performed using Qubit ssDNA Assay Kit (Thermo Fisher Scientific) and Agilent Bioanalyzer 2100. All libraries were further sequenced by the DIPSEQ T1 sequencing platform generating reads containing 30‐bp read 1 (including the 10‐bp cell barcode 1, 10‐bp cell barcode 2, and 10‐bp unique molecular identifier (UMI)), 100‐bp read 2 and 10‐bp barcodes (sample index) [19].

### 2.10 Preparation of scRNA‐Seq Data

Sequencing data filtered, and gene expression matrix was obtained using DNBelab C Series scRNA analysis‐software. Briefly, all samples were performed sample de‐multiplexing, barcode processing, and single‐cell 3′ unique molecular identifier (UMI) counting with default parameters. Processed reads were then aligned to GRCh38 genome reference using STAR (v2.5.3) [20]. Valid cells were automatically identified based on the UMI number distribution of each cell by using the “barcodeRanks” function of the DropletUtils tool to remove background beads and the beads that had UMI counts less than the threshold value. Finally, we used PISA to calculate the gene expression of cells and create a gene cell matrix for each library.

### 2.11 Analysis of scRNA‐Seq Data

The PCA (principal component analysis) was performed using the Seurat R Package to identify the first 10 principal components in the feature‐barcode matrices for dimensionality reduction [21]. Following this, the t‐distributed stochastic neighbor embedding (t‐SNE), and the uniform manifold approximation and projection (UMAP) algorithm were employed for visualization of the reduced data in two‐dimensional space and expression similarity analysis, respectively. The data visualization was carried out by Loupe Cell Browser Software (v3.1.0) and Seurat for clustering, heatmap generation, and differential gene expression analysis.

### 2.12 Differential Expression Analysis and Cell Type Identification

To identify different expressed genes (DEGs) within each cluster, the Seurat function FindAllMarkers function was used to find genes that are more highly expressed in that cluster compared to the rest of the clusters. The log2 fold change (L2Fb) was used as an estimate of the log2 ratio of mean gene expression in a cluster to that in all other clusters and cells, based on a negative binomial test. The *p*‐value of the output data reported here had been adjusted for multiple testing using the Benjamini–Hochberg procedure. Cell type annotation was mainly identified based on the marker genes from previous studies and the CellMarker database [22]. The sample dates of cells were assigned to marker genes within each cell type for validating the cell type assignments.

### 2.13 Gene Enrichment and Pathway Analysis

In this study, we conducted the following differential expression comparisons: (1) the DEGs between one cell type and the remaining cell types for identifying cell‐type marker genes; (2) the DEGs between AAO and Sham control samples for identifying disease‐associated changes at the cell type level; (3) the DEGs between one subcluster of a given cell type and the remaining subclusters of the same cell type for determining subcluster‐specific genes. All these DEGs mentioned above served as input for gene enrichment analysis via Metascape, a web‐based bioinformatics portal for gene annotation and analysis resource that especially supports meta‐analysis of multiple gene lists [23]. To perform gene enrichment analysis, we first identified all statistically enriched terms (GO Biological Processes, KEGG Pathway, and Reactome Gene Sets), and calculated accumulative hypergeometric *p*‐values and enrichment factors for filtering.

### 2.14 Hierarchical Clustering and Enrichment Analysis

We executed hierarchical clustering of significant terms into a tree based on Kappa‐statistical similarities among their gene patterns, and the tree was segmented into term clusters at a threshold (0.3 kappa score). Given that a Circos plot provides a more intuitive and scalable representation compared to a Venn diagram, we employed the former to illustrate the overlap among the gene lists. Genes were interconnected in the network if they shared the same ontology term, which was significantly enriched in both input gene lists. To prevent linking genes based on very general annotation, functional overlaps were computed based on ontology terms that contain fewer than 100 genes. For cell type–specific DEGs between AAO and Sham groups, we performed the enrichment analysis using online resources such as Enrichr [24].

### 2.15 Trajectory Analysis

The genes were input for trajectory analysis via the Monocle2 R package, and dimensionality reduction was conducted by applying the DDRTree method with default parameters [25]. The branch expression analysis modeling (BEAM) implemented in Monocle 2 was used to identify specific genes that are enriched along particular branches in the pseudotime tree. Branched heatmaps were created with the significant branch‐specific expression genes (*q* value < 5 × 10^−5^). The orderCells function was analyzed by using the root state argument to specify the homeostatic Mon/Mφ branch as the start point of the trajectory. In addition, CytoTRACE was used to determine the recommended differentiation ability of cells [26].

### 2.16 Cellular Interactions Analysis

Molecular interactions between the cells were identified by the recently developed CellPhoneDB. Normalized and filtered scRNA‐seq data with the clusters previously identified by Seurat were used for CellPhoneDB analysis [27] and CellChat [28]. Since the current CellPhoneDB release only accepts human ensembl IDs as input, murine ensembl IDs were converted to human ensembl IDs using biomaRt. The ligand–receptor pair was included in the analysis only when the percentage of cells expressing the receptor and ligand genes exceeded 30%. For the polymeric complexes, the expression value of the subunit with lower average expression was selected as the expression of the receptor for subsequent statistical analysis. This software could also identify the cell type–specific ligand–receptor interactions according to their priority. Then, pair comparisons were performed between all cell types in the data set. Initially, the cell type markers of all cells were randomly arranged 1000 times to form a new cell cluster, and the average expression level of the ligand in the randomly arranged cell cluster and the average expression level of the receptor in the interacting cell clusters were calculated. Finally, a zero distribution is generated for each ligand–receptor pair in each pair comparison between the two clusters.

### 2.17 Immunofluorescence Staining

To compare astrocyte and oligodendrocytes precursor cell (OPC) numbers between two groups, we used immunofluorescence staining with GFAP for astrocytes and NG2 for OPC. The brain tissue paraffin coronal sections were cut into 4.5 μm. Tissue sections were dewaxed and underwent antigen retrieval with Tris‐EDTA (pH 9.0) for 15 min. Primary antibodies were mouse anti‐GFAP (1:300, Santa Cruze, USA) and rabbit anti‐NG2 (1:300, Millipore, USA). The secondary antibodies were Alexa Fluor 488‐,594‐conjugated donkey anti‐rabbit antibody (1:300; Invitrogen, USA). The tissue slice panoramic scanning system (Pannoramic MIDI, 3DHistech Company, Hungary) was used to take photos.

### 2.18 Statistical Analysis

All statistical analysis was described in the figure and data are presented as mean ± SD. Shapiro–Wilk test was applied for the evaluation of normality for each set of data compared. For normally distributed populations of data points, a two‐tailed unpaired Student's *t*‐test was used for comparisons between two groups. For data that failed the normality test, a Mann–Whitney test (2 groups), or a Kruskal–Wallis test with a Dunn post‐test (> 2 groups). *p* < 0.05 were usually considered to be statistically significant. The threshold for different expressed genes (DEGs) is *p*‐value < 0.05 and abs(log2(Fold Change)) > 0.4.

## 3 Results

### 3.1 AAO Induced Neurological Function Impairment and Pathological Damage in Mice

In this experiment, the C57BL/6 mouse was used to establish the AAO model (Figure 1A). Firstly, the change in body weight of mice was monitored weekly post‐modeling (Figure 1B). After modeling, the animals' body weight experienced an initial decrease in first week but showed an increase in 2–4 weeks. Compared to the Sham group, the AAO group exhibited a significant decreased in body weight during the 28‐day period.

Subsequently, to assess the effect of AAO on the neurological function of mice, behavioral tests including rotarod, Y‐maze, and new object recognition tests were conducted on the 28th day after AAO modeling. As shown in Figure 1C, there were no differences in rotarod performance between the Sham group and AAO group. However, in the Y‐maze test, the time spent in the new arm was significantly lower in the AAO group compared to the Sham group (Figure 1D). Similarly, in the novel object recognition test, the recognition index percentage was significantly lower in the AAO group compared to the Sham group (Figure 1E). These results revealed that AAO does not affect motor coordination and balance but impairs cognitive function and recognition memory Since the Y‐maze and novel object recognition tests assess spatial memory, cognitive function, and recognition memory, both closely linked to the hippocampus (Schizophrenia‐Like Behaviors Arising from Dysregulated Proline Metabolism Are Associated with Altered Neuronal Morphology and Function in Mice with Hippocampal PRODH Deficiency), we speculate that the AAO model primarily damages hippocampal tissue.

To further determine the extent of damage in AAO mice, we employed H&E staining to observe the pathological injury in brain tissue at 28 days after reperfusion. The results showed an increase in pyknotic nuclei in the neurons of the CA1 and DG regions of the hippocampus, indicating an increase in damaged cells (Figure 1F,G). Compared to the Sham group, there was a notable increase in the percentage of damaged neurons in the hippocampus of the AAO group. Although AAO‐induced ischemia is known to cause hippocampal damage, the specific cellular and molecular responses are not well understood. This study employs scRNA‐sequencing to reveal cell type–specific gene expression changes and interactions critical to the injury and recovery processes.

### 3.2 Identification of Major Cell Types in AAO Injury

**FIGURE 2:**
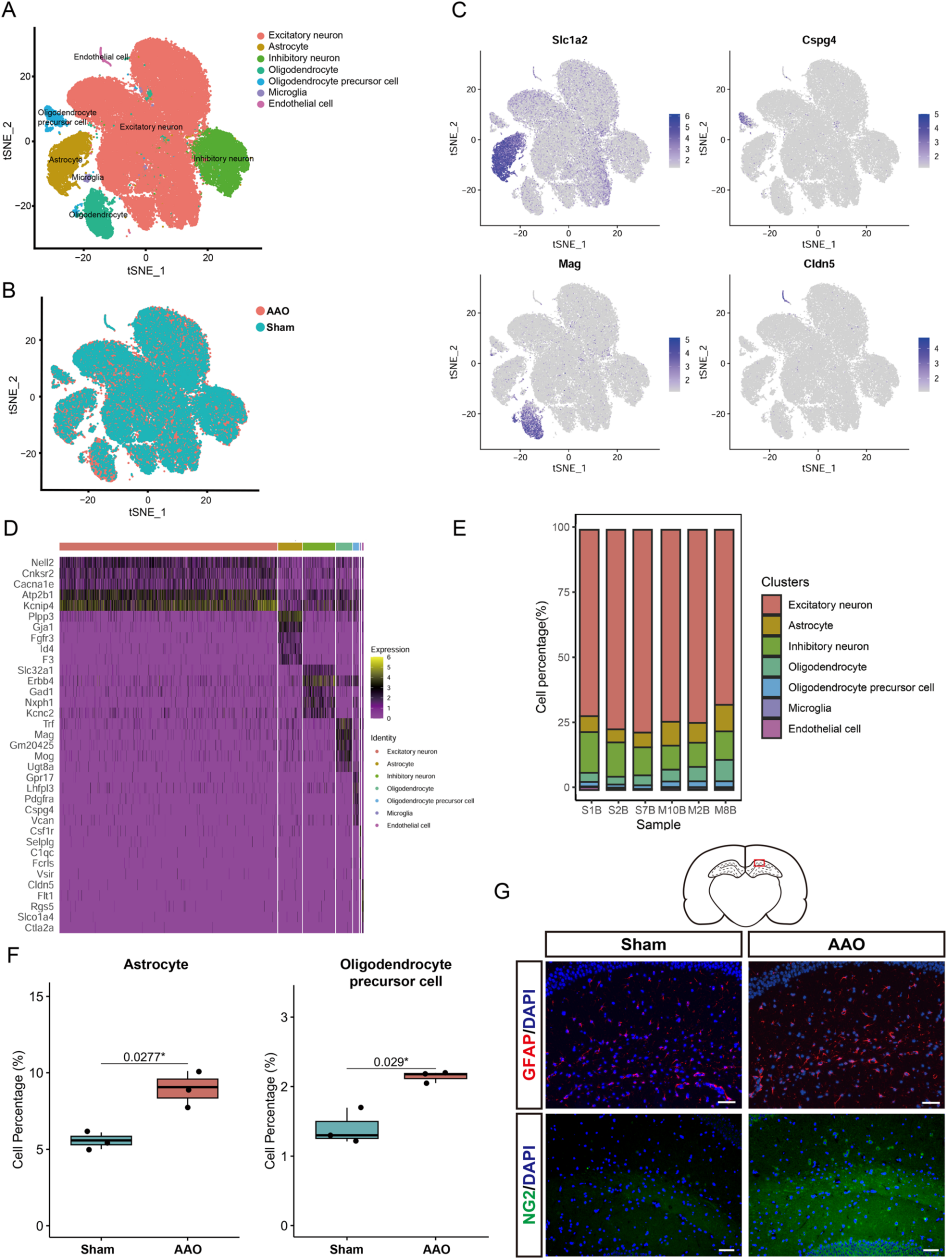
Comprehensive landscape of scRNA‐seq reveals cells‐type proportion differences between Sham and AAO groups in hippocampus tissue. (A) t‐SNE plot showing clusters of single cells colored by cell types. The dots represent the cells and are colored to represent distinct cell types, including Excitatory neurons (red), Inhibitory neurons (green), Astrocytes (blue), OPC, (yellow), MicrogliA, (brown), and Endothelial cells (pink). (B) t‐SNE plot which compares cellular compositions between two groups (AAO and Sham). The dots represent the cells and are colored to represent these two conditions. (C) t‐SNE plots showing the expression distributions of selected marker genes including *Slc1a2*, *Cspg4*, *Mag*, and *Cldn5* across different cell types. (D) Heatmap displaying gene expression levels of the top 5 marker genes across identified cell clusters or types. The color gradient ranges from purple (low expression score) to yellow (high expression score). (E) the proportions of each cell type under different sample conditions (Sham and AAO). (F) Box plots compares proportion difference for Astrocytes and OPC, between Sham and AAO groups. *p < 0.05 by unpaired two‐tailed *t*‐test. (G) Representative immunofluorescence images of brain tissue section. The astrocyte cells were labeled with GFAP as red, the OPC, were labeled with NG2 as green, and the nuclei were labeled with DAPI as blue. Scale bar = 50 μm. Data are presented as mean SEM, *n* = 3. *p < 0.05.

To mitigate potential bias stemming from differential expressed cell surface markers in specific populations, we isolated all single cells from the hippocampus of both the AAO and Sham groups. We obtained a total of 62,624 cells, with an average of 1295 genes per cell and an average of 2610 unique molecular identifiers (UMIs) per cell. Using t‐SNE plot analysis after batch‐effect correction, we observed distinct clustering of individual cells from both the AAO and Sham groups. By detecting known cell type markers, we identified seven transcriptionally distinct clusters, including microglia, endothelial cells, astrocytes, oligodendrocytes, OPC, excitatory neuron cells, and inhibitory neuron cells (Figure 2A). The cellular distribution of two groups were shown in Figure 2B.

The validity of cell clustering in this study was confirmed by the majority of previously published marker genes for each cell type. Additionally, we presented four selected marker genes specific to four cell clusters, including, *Slc1a2*, *Cspg4*, *Mag*, and *Cldn5* (Figure 2C). It was also presented for the selected marker gene of neuron (Figure S1A), excitatory neuron (Figure S1B), and inhibitory neuron (Figure S1C). The top 5 most highly expressed genes within each type relative to the rest of cell types were shown in (Figure 2D). To identify hippocampus cell types susceptible to AAO, we conducted a comprehensive comparison of the composition proportions between each cell type in the Sham and AAO groups. We observed the percentage of seven cell types in the AAO samples compared to the Sham samples (Figure 2E). Furthermore, the comparative analysis revealed a significant increase in the proportions of astrocytes and OPC following AAO (Figure 2F). Meanwhile, to further confirmed the regulatory effect of AAO model on these cell types, we used the GFAP to label astrocytes and NG2 to label OPC. As shown in Figure 2G and Figure S2A,B compare with the Sham group, the expression level of GFAP and NG2 proteins was significantly upregulated in AAO group. These increase means the association between the key characteristics of the increased cell types and the AAO injury.

### 3.3 Cell Type–Specific Gene Expression and Overall Assessment of DEGs and Pathways Associated With AAO Injury

To investigate the DEGs and the pathways or biological processes involved in the pathogenesis of AAO injury within each identified cell type, we compared the significant gene changes between the AAO and Sham groups, and summarized the top representative pathways and processes enriched from each cell‐subset specific DEGs (Figure S3 and Table S1).

Most strikingly, a total of 115 DEGs (*p*‐value < 0.05 and abs(log2(Fold Change)) > 0.4) between AAO and Sham samples were identified in endothelial cells, ranking endothelial cell at the top of the list (Figure S3). This finding suggested that endothelial cells may experience the direct stress and damage after AAO injury of the hippocampus. This change may affect the delicate balance between maintaining brain homeostasis and allowing necessary exchanges with the bloodstream. The astrocyte had 27 unique DEGs (*p*‐value < 0.05 and abs(log2(Fold Change)) > 0.4) among all cell types, ranking at the send of the list, followed by excitatory and inhibitory neuron. Oligodendrocytes and OPC shared the highest proportion of common DEGs (*p*‐value < 0.05 and abs(log2(Fold Change)) > 0.4) post‐AAO among all cell types. These cells are known to play significant roles in supporting function of central nervous system [29, 30, 31].

**FIGURE 3:**
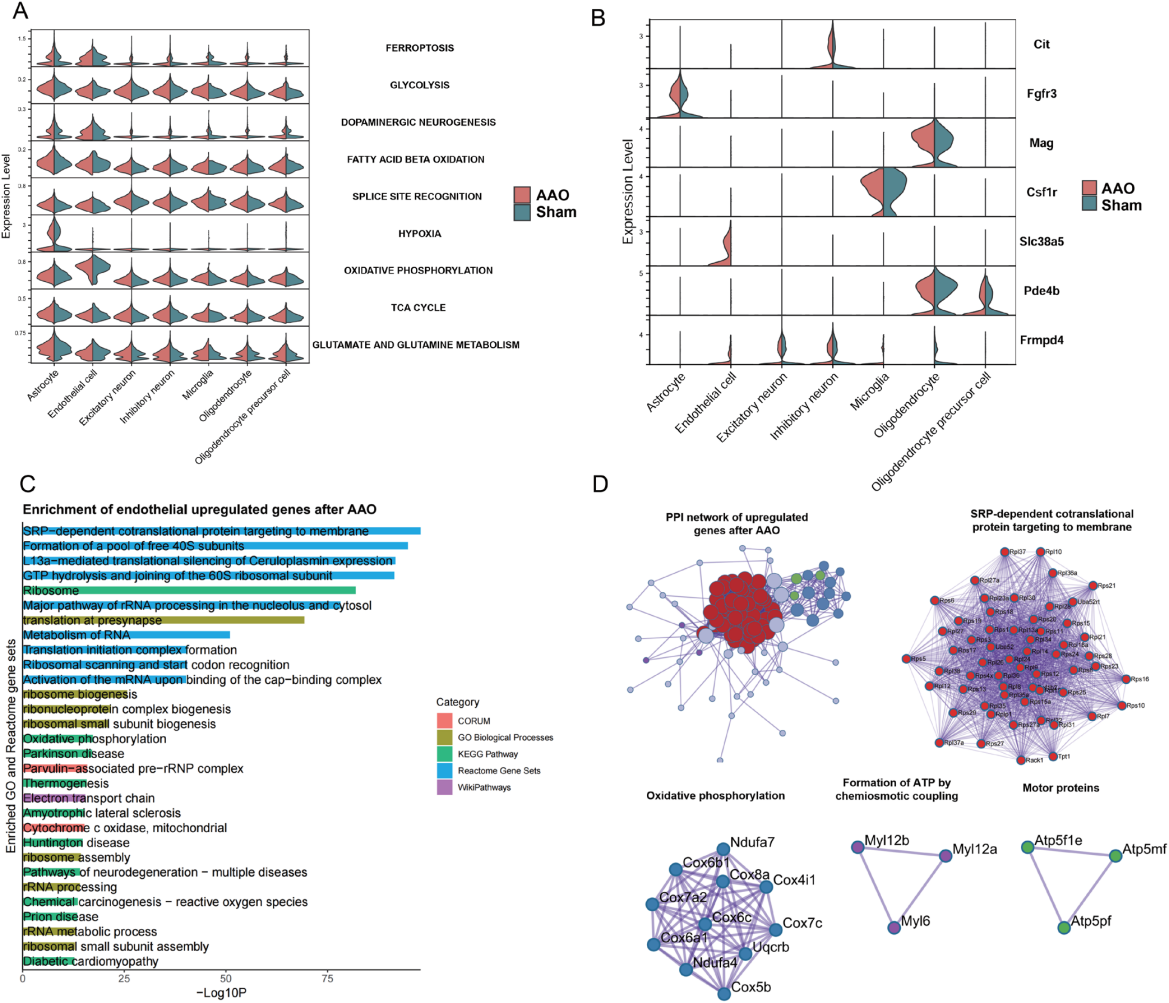
The differential expression analysis of each cell type between Sham and AAO groups. (A) Violin plots illustrating the GSVA, scores of several pathways across different cell types and groups. Each row corresponds to a specific pathway, including FERROPTOSIS, GLYCOLYSIS, DOPAMINERGIC, NEUROGENESIS, FATTY ACID BETA, OXIDATION, SPLICE SITE RECOGNITION, HYPOXIA, OXIDATIVE PHOSPHORYLATION, TCA, CYCLE, and GLUTAMATE AND GLUTAMINE METABOLISM. The color gradient ranges from blue (low GSVA, score) to red (high GSVA, score). (B) Features violin plots showing the shared‐brain DEGs between the Sham and AAO groups. The *y*‐axis lists specific genes, and colors differentiate the two groups. (C) Enrichment Pathways for Endothelial Upregulated Genes This bar graph illustrates the enrichment pathways for genes that are upregulated in endothelial cells. The length of each bar represents the significance level of the enrichment, with longer bars indicating higher significance. Different colors are used to denote various gene set categories, allowing for easy visual differentiation between the pathways. (D) Protein–Protein Interaction (PPI) Analysis Results for Endothelial Upregulated Genes Post‐AAO. This network diagram shows the results of the PPI analysis for genes that are upregulated in endothelial cells following AAO (Acute Aortic Occlusion). Each node in the network represents a gene, and the nodes are color‐coded to distinguish between different gene clusters. The connections between nodes indicate interactions between the proteins encoded by these genes, highlighting the complex interplay within the endothelial cell environment post‐AAO.

Moreover, expression level analysis for several critical pathways between AAO and Sham group were assigned to the distinct cell types (Figure 3A). Elevated expression scores applied by Seurat add‐module function in AAO suggest increased susceptibility to FERROPTOSIS, a form of regulated cell death associated with lipid peroxidation and iron metabolism. It can also be seen that DOPAMINERGIC_NEUROGENESIS mainly occurs in astrocytes and epithelial cells, while alternative splicing generally appears to increase after AAO injury in all cell types additional pathways, such as glycolysis, fatty acid metabolism, and hypoxia, also show varying scores. Among these, the HYPOXIA pathway exhibits the highest expression in astrocytes, while OXIDATIVE PHOSPHORYLATION shows increased expression levels in astrocytes, OPC, and oligodendrocytes after AAO injury. These pathways reveals that these cells adapt to environmental stress after injury by activating different biological function, participating in the processes of cellular protection or repair.

To explore potential molecular markers for the diagnosis and treatment of AAO injury, we aimed to identify unique and shared DEGs within each cell type between the Sham and AAO groups (Figure 3B). In our experiments, we observed a specific upregulation of the *Fgfr3* (Fibroblast Growth Factor Receptor 3) encoding a receptor for fibroblast growth factors [32]. It has been reported that *Fgfr3* are required for vertebrate astrocyte morphogenesis. *Slc38a5* (Solute Carrier Family 38 Member 5) is involved in amino acid transport and had been upregulated in endothelial cell specially [33]. It regulates endothelial cell glutamine uptake and vascular growth. *Cit* is involved in cell division and cytokinesis and had higher expression in inhibitory neuron, *Mag* is only upregulated in oligodendrocyte, and it was demonstrated that myelin‐associated glycoprotein (MAG) was a novel receptor for angiopoietin‐like protein 2 (ANGPTL2) [34]. The binding of ANGPTL2 to MAG efficiently promoted the differentiation of oligodendrocytes. This observation may indicate there is more differentiation of oligodendrocytes after AAO injury. In Figure 3C, the enrichment pathways for endothelial upregulated genes are presented. These pathways include Metabolism of RNA, Electron transport chain, and Pathways of neurodegeneration. This suggests that these specific pathways are significantly enriched in the upregulated genes, highlighting their potential roles in endothelial function. In Figure 3D, the results of the protein–protein interaction (PPI) analysis for endothelial upregulated genes after AAO are shown. The identified modules include SRP‐dependent co‐translational protein targeting to membrane, oxidative phosphorylation, formation of ATP by chemiosmotic coupling, and Motor proteins. This indicates that these specific gene clusters may have distinct roles in the response to AAO, providing insights into the molecular mechanisms underlying this process. Taken together, this study provided a context for a comprehensive understanding of cell type–specific DEGs associated with AAO injury, as well as related pathways or biological processes in the hippocampus following the injury.

### 3.4 AAO Injury Leads to Specific Cell–Cell Communication Patterns in the hippocampus

The responses triggered by AAO involve intricate interactions among hippocampus cells, including adhesion molecules on cell surfaces and chemical signaling molecules. In this study, we employed the CellPhoneDB database to infer potential cell–cell communication in the hippocampus and examine its variation during AAO. We observed both consistent and distinct interactions patterns between different cell types, including incoming strength in Sham (Figure S4A) and AAO (Figure S4B) and outcoming strength in Sham (Figure S4C) and AAO (Figure S4D).

**FIGURE 4:**
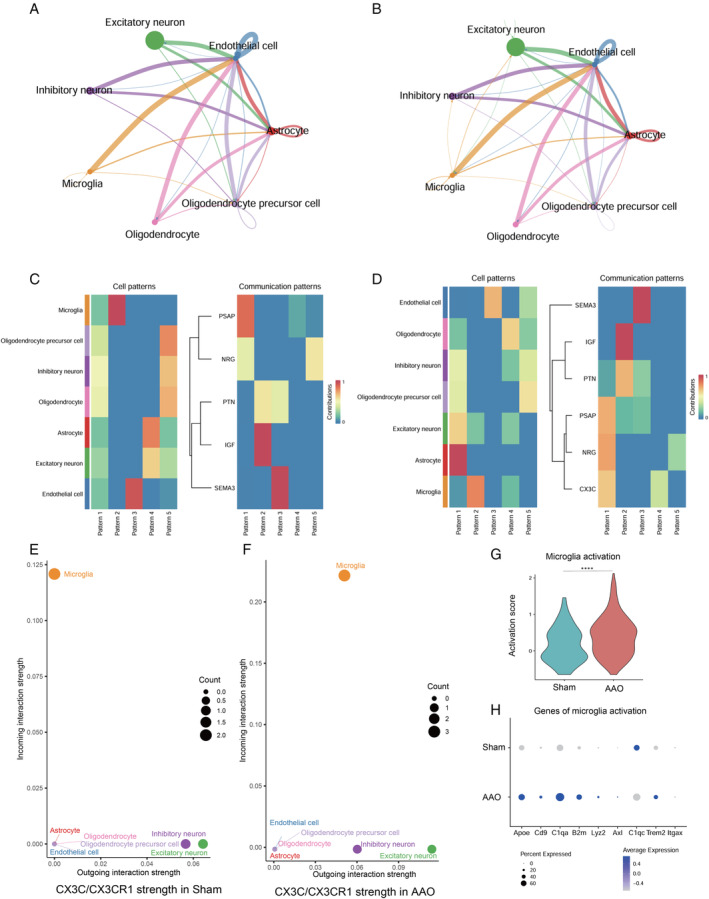
Comparative cell–cell interactions in Sham and AAO mice. (A, B) Network diagram showing interactions between various cell types in Sham (A) and AAO (B) group. Color‐coded lines denote communication interaction, and the line width represents the interaction strength (C, D). Heatmap depicting communication patterns in Sham (C) and AAO (D) groups. Intensity of colors reflects the strength of interactions. Cool colors (blue to green) indicate the weaker interactions; warm colors (yellow to orange) indicate the stronger interactions. Scatter plot illustrating ongoing interaction strengths against incoming interaction strengths for CX3C, ligand–receptor pair in Sham (E) and AAO (F) groups of the cell types. Point size corresponds to interaction count. (G) Violin plots depicting microglia activation scores demonstrate significantly increased activation in the AAO group compared to Sham. ****p < 0.0001 by unpaired two‐tailed *t*‐test. (H) Dot plot representation of genes associated with microglia activation in terms of both percent expressed (dot size) and average expression (dot color intensity) in the AAO group relative to Sham.

The cell–cell interaction landscape in the hippocampus was shown in the Sham (Figure 4A) and AAO groups (Figure 4B) respectively. It can been seen that, in the AAO groups, there are new inferred communications between microglial and other cells, particularly, interactions involving microglia and inhibitory neurons and excitatory neurons. The new interactions between microglia and endothelial cells are also predicted.

Cell–cell communication analysis in the Sham controls revealed abundant growth factor signaling pathways, including PSAG, NRG, PTN, IGF, and SEMA3 (Figure 4C). Unique interactions involving CX3C associated with AAO were found (Figure 4D). CX3C refers to a chemokine receptor called CX3CR1. It is known to CX3CR1 deficiency limits the expansion and activation of microglia and macrophages within the CNS and leads to protection of neurological function [33, 34, 35]. The CX3C/CX3CR1 incoming and outcoming strength were shown in the Sham (Figure 4E) and AAO group (Figure 4F) respectively. CX3C/CX3CR1 strength mainly increased in microglia after AAO suggesting potential neuroprotective effects through signaling pathways in microglia. We scored the activation levels of microglia using genes previously shown to be specific for activated microglia [15]. Cells for AAO showed significantly higher activation score, as indicated by Figure 4G. It was illustrated that the expression levels of all the various genes associated with microglia activation were higher in AAO except *C1qc* (Figure 4H).

Overall, these intercellular interactions indicate a close relationship between peripheral cell dynamics and molecular features of hippocampus resident cells during AAO injury. These findings have implications for understanding the prognosis and therapeutic responses in AAO.

### 3.5 Unique Gene Expression of Astrocyte Cells Enriched in Positive Regulation of Neurogenesis and Showed Alterations mRNA Processing After AAO

**FIGURE 5:**
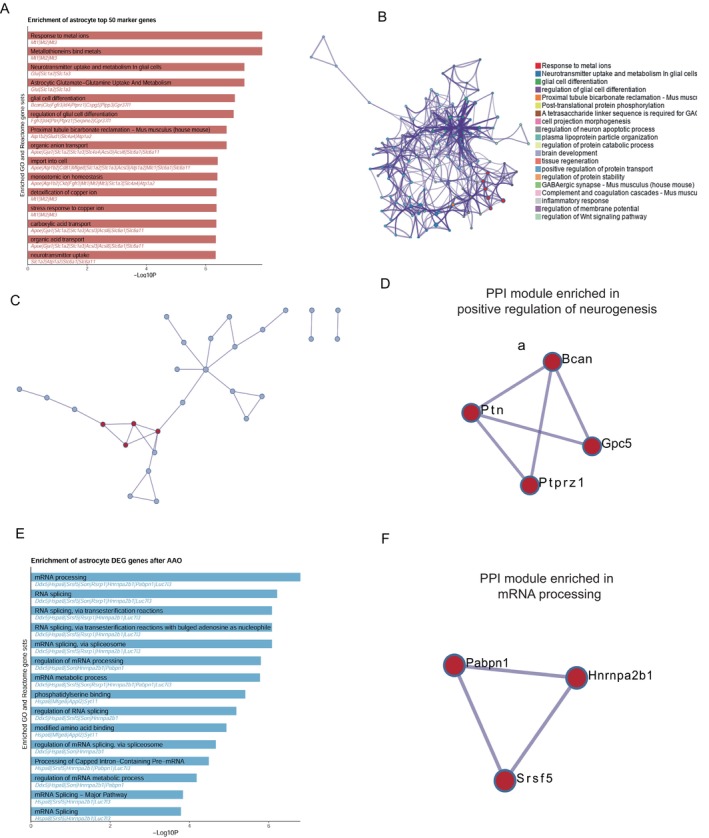
Enrichment and PPI analysis of astrocyte marker genes and different expressed genes between groups. (A) Enrichment pathways for astrocyte marker genes. The color intensity in this bar graph corresponds to the significance level of enrichment, with darker colors indicating higher significance. (B) Network diagram illustrating connections between various enriched pathways. Different colors represent distinct pathway categories. (C) PPI analysis results for astrocyte marker genes. Nodes are colored differently to distinguish between different gene clusters. (D) Detailed view of one module from panel C, highlighting specific genes involved in positive regulation of neurogenesis. (E) Bar graph representing DEGs enrichment in astrocytes. Darker bars signify higher significance levels. (F) Expanded view of another module from the PPI analysis of the DEGs, focusing on mRNA, processing.

In this study, we focused on specific markers of astrocyte cells compared to other cell types and their enrichment results mainly related to regulation of neural function, including neurotransmitter uptakes, regulation of glial cell differentiation, astrocytic glutamate‐glutamine uptake and metabolism, and so on (Figure 5A). We then constructed gene enrichment networks based on each unique marker genes (Figure 5B).

Notably, the PPI analysis revealed a positive regulation of neurogenesis associated with these markers (Figure 5C,D). In this module, *Bcan* (Brevican) is an extracellular matrix proteoglycan that plays a role in neuronal plasticity. It is involved in cell adhesion, migration, and axon guidance. *Bcan* influences the formation and maintenance of neural circuits by modulating cell–cell interactions and synaptic plasticity. *Ptn* (Pleiotrophin) is a growth factor that promotes neuronal survival, differentiation, and axon outgrowth, which is crucial for the development of the nervous system, including neurogenesis [35, 36]. It enhances the production of new neurons and contributes to learning and memory processes. *Gpc5* (Glypican 5) are cell surface heparan sulfate proteoglycans. *Gpc5* is involved in cell signaling, including *Wnt* pathways, and likely influences neurogenesis by modulating signaling pathways critical for neural stem cell proliferation and differentiation. *Ptprz1*(Protein Tyrosine Phosphatase Receptor Z1) is a receptor‐type protein tyrosine phosphatase. It regulates cell signaling by dephosphorylating tyrosine residues. *Ptprz1* is implicated in differentiation and myelination and may impact neural precursor cell behavior [37, 38]. It has been shown that the proportion of astrocytic cells increased after AAO, leading to an elevated function of modules and genes above in astrocyte after AAO injury.

Furthermore, we investigated the upregulated genes enrichment pathways in astrocytic cells after surgery (Figure 5E), which were manly about mRNA processing, phosphatidylserine binding, and so on. Through PPI analysis, we identified a module related to mRNA processing, indicating that these significant genes are involved in the mRNA processing pathway and exhibit protein interactions (Figure 5F). In this module, *Pabpn1* (Poly(A) Binding Protein Nuclear 1) is a multifunctional regulator of mRNA processing. *Pabpn1* regulates alternative polyadenylation site (PAS) utilization, impacting cell and tissue function. *Hnrnpa2b1* (Heterogeneous Nuclear Ribonucleoprotein A2/B1) is involved in RNA splicing and other aspects of mRNA processing. It regulates the alternative splicing of specific genes, including BIRC5, which plays a role in growth and metastasis. *Hnrnpa2b1*'s actions impact gene expression patterns and cellular functions. *Srsf5* (Serine/Arginine‐Rich Splicing Factor 5) is a member of the serine/arginine‐rich (SR) protein family involved in pre‐mRNA splicing. *Srsf5* modulates splice site selection, ensuring accurate mRNA processing. It interacts with other splicing factors to regulate exon inclusion or exclusion, ultimately affecting protein diversity and function.

In summary, astrocytic cells play a crucial role in positively regulating neurogenesis and mRNA splicing pathways after AAO.

### 3.6 Heterogeneity of Astrocytes During AAO Injury

**FIGURE 6:**
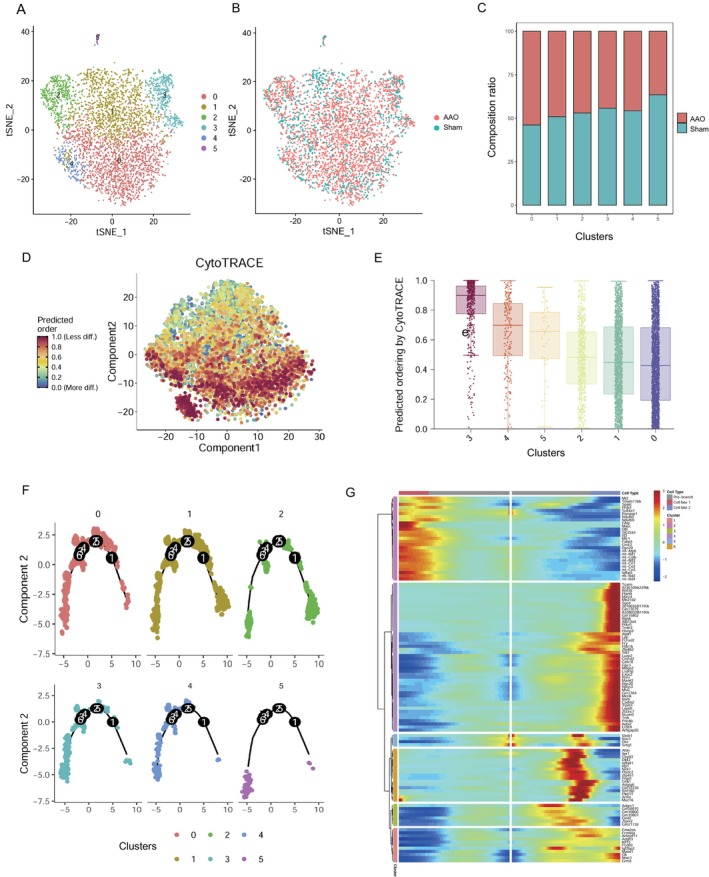
Analysis of astrocytes subclusters. (A) t‐SNE plot clustering of individual cells, color‐coded to represent distinct subgroups of astrocytes. Colors indicate different clusters. (B) t‐SNE plot which compares cellular compositions between two groups (AAO and Sham). The dots represent the cells and are colored to represent these two conditions. (C) Bar graph showing cell composition ratio across different clusters. (D) Scatter plot with CytoTRACE score, indicating cell maturity. (E) Boxplot of CytoTRACE score across the clusters showing differences in developmental stages. (F) Pseudotime analysis of astrocytes using Monocle based on Seurat‐based clusters. (G) Heatmap of differentially expressed genes, ordered based on their similarity dynamic, trend following pseudotime process.

To investigate the heterogeneity of astrocytes in the context of AAO injury, six clusters were identified (Figure 6A) and cellular compositions between two groups (Figure 6B). Clustering tree has been produced directly from Seurat objects of astrocyte (Figure S5A). The composition ratio of the various astrocyte subclusters was altered by the AAO injury. Among these clusters, cluster0 represented the most astrocytes the hippocampus from the AAO group (Figure 6C). The CytoTRACE analysis were implied to the clusters (Figure 6D), which could indicate cell maturity or developmental stages. Figure S5B displays the correlation of various genes with CytoTRACE. It showed that the cluster 0 persists the highest level of cell maturity and the clusters are correlated to the developmental stages (Figure 6E). This indicates that the development of astrocyte subclusters was altered by the AAO injury and showed more likely to be differentiated in AAO group.

Furthermore, we employed the BEAM algorithm in Monocle 2 to identify the different cell states and their association with the AAO‐induced insult. Each dot in the analysis represented a single‐cell expression profile ordered in “pseudotime,” and the connecting lines between the dots depicted the trajectory of relatedness in their expression patterns (Figure 6F). The pseudotime analysis based on Seurat‐defined astrocytes subclusters revealed a distinct differentiation trajectory. The cluster 5 followed a separate trajectory branch. The subclusters were distributed across the remaining branches. To track the changes in gene expression during different astrocytes fates, we examined the fluctuation of differentially expressed genes along the pseudotime order (Figure 6G).

### 3.7 Gene Signature Expression of OPC Exhibit Enhanced Synapse Organization, Assembly, and Maturation

**FIGURE 7:**
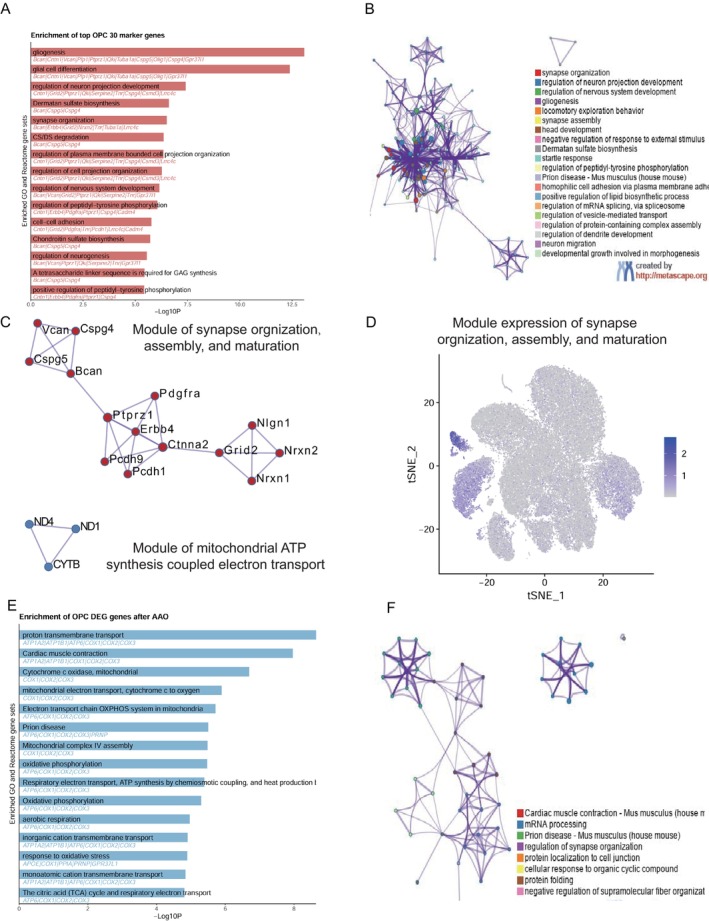
Enrichment and PPI analysis of OPC, marker genes and different expressed genes between groups. (A) Enrichment pathways for OPC, marker genes. The color intensity in this bar graph corresponds to the significance level of enrichment, with darker colors indicating higher significance. (B) Network diagram illustrating connections between various enriched pathways. Different colors represent distinct pathway categories. (C) Detailed view of one module from PPI analysis results for OPC, marker genes. Nodes are colored differently to distinguish between different gene clusters, highlighting specific genes involved in synapse organization, assembly, and maturation. (D) Dim plots showing the expression level of the module in panel C across cell types. The color intensity signifies expression levels, indicating higher expression. (E) Bar graph representing DEGs enrichment pathways in OPC. Darker bars signify higher significance levels. (F) Network diagram illustrating connections between various enriched pathways in panel E. Different colors represent distinct pathway categories.

In this study, we focused on specific markers of OPC compared to other cell types and their enrichment results mainly related to regulation of neural functions, including gliogenesis, synapse organization, and regulation of nervous system development (Figure 7A). We then constructed gene enrichment networks based on each unique marker genes (Figure 7B).

Notably, the PPI analysis revealed a module of synapse organization, assembly, and maturation associated with these markers (Figure 7C). The expression of this module is highly expressed in OPC cluster (Figure 7D). In this module, *Cspg5* (Chondroitin Sulfate Proteoglycan 5) protein may function as a neurotrophic and differentiating factor, playing a role in neuronal development and function. *Bcan* (Brevican) is an extracellular matrix protein involved in synaptic formation and neuronal connectivity. *Pdgfra* (Platelet‐Derived Growth Factor Receptor Alpha) protein is a receptor tyrosine kinase involved in neuronal development and synapse formation. *Ptprz1* (Protein Tyrosine Phosphatase Receptor Type Z1) negatively regulates the proliferation of glial precursor cells in the embryonic spinal cord. It is also important for the proper differentiation of mature myelinating glial cells. *Erbb4* (Erb‐B2 Receptor Tyrosine Kinase 4) is a receptor tyrosine kinase associated with neuronal development and synaptic function. *Nign1* (Neuronal Immunoglobulin Superfamily Member 1) is a neuronal‐specific immunoglobulin superfamily member, likely involved in cell adhesion and synaptic organization.

Furthermore, we investigated the function of upregulated gene in OPC cells after surgery, which is mainly enriched in proton transmembrane transport, mitochondrial electron transport, response to oxidative stress (Figure 7E). The enrichment networks based on these genes are shown in Figure 7F. In summary, the increased proportion of OPC and the elevated enrichment of these markers in pathways and modules suggest that OPC exhibit enhanced synapse organization, assembly, and maturation following the procedure.

### 3.8 Gene Expression Analysis of OPC Subtypes in AAO Injury

**FIGURE 8:**
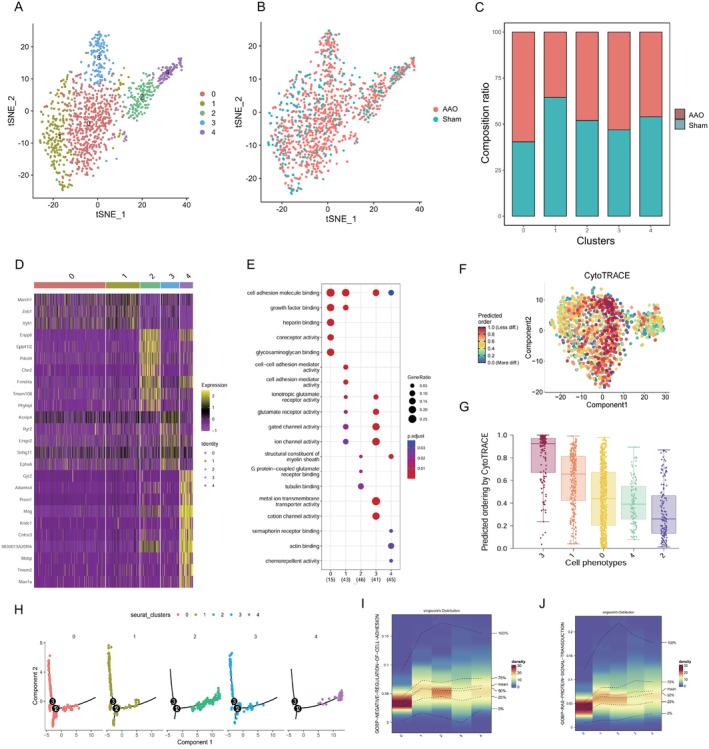
Analysis of OPC, clusters. (A) t‐SNE plot clustering of individual cells, color‐coded to represent distinct subgroups of OPC. Colors indicate different clusters. (B) t‐SNE plot which compares cellular compositions between two groups (AAO and Sham). The dots represent the cells and are colored to represent these two conditions. (C) Bar graph showing cell cluster proportion across different samples. Each color corresponds to a, specific cluster. (D) Heatmap displaying average expression and percent expressed of selected marker genes in each cluster. Color intensity reflects expression levels. (E) Gene Oncology enrichment of the marker genes of each cluster. Color gradients represent the significance. (F) Scatter plot with CytoTRACE score, indicating cell maturity. (G) Boxplot of CytoTRACE score across the clusters showing differences in developmental stages. (H) Pseudotime analysis of astrocytes using Monocle based on Seurat‐based clusters. Gene Expression Density: Density plot illustrating significant distribution differences in gene expression of the GOBP‐NEGATIVE‐REGULATION‐CELL‐ADHESION (I) and GOBP‐RAS‐PROTEIN‐SIGNAL‐TRANSDUCTION (J). Color gradients denote density levels.

OPC are essential cells in the hippocampus that play a critical role in various physiological and pathological processes. Recent studies utilizing scRNA‐seq OPC cluster 2 have uncovered the heterogeneity of OPC in development, homeostasis, and diseases. In this study, we identified five distinct OPC clusters (Figure 8A) and cellular compositions between two groups (Figure 8B). Clustering tree has been produced directly from Seurat objects of astrocyte (Figure S6A), suggesting 5 cluster. Based on composition ratio, OPC cluster 0 (OPC0) primarily consisted of cells from the control group, whereas three clusters (OPC2, OPC3, and OPC4) were predominantly composed of cells from the AAO group. OPC1 contained higher proportion of cells from both the control (Figure 8C).

Notably, the most AAO‐associated subcluster OPC1 and OPC2 shared a set of significant genes, including *Cspg5*, *Pdgfra*, *Apoe*, *Atp1a2*, and *Serpine2* (Figure 8D). OPC2 expressed a set of genes associated with neurodegeneration, included *Enpp6*, *Epb41l2*, *Pdcd4*, *Fyn*, and *Chn2* (Figure 8D). The enriched GO functions of upregulated genes within each cluster after AAO injury were shown in Figure 8E. OPC1 showed exclusive upregulation of ion channel activity and glutamate receptor activity compared to OPC0. In addition, a cytoTRACE cell differentiation ability analysis was performed, including dot plots (Figure 8F). Figure S6B displays the correlation of various genes with CytoTRACE. The results showed that OPC0 has a higher differentiation ability than OPC1 (Figure 8G). Furthermore, a pseudotime analysis was conducted (Figure 8H), showing that OPC0 is in the later stage of the sequence. Through GSVA cell pathway function scoring, it was observed that OPC0 showed significant differences in cell adhesion (Figure 8I) and protein signal transduction (Figure 8J) compared to other clusters.

These results indicate that after AAO injury, there are changes in the subgroup composition of OPC, especially an increase in the proportion of OPC0 and a decrease in the proportion of cluster 1. This may reflect the cells' response to injury and their role changes during recovery. In addition, cells in cluster 0 have a higher differentiation ability, which may be due to their important role in the recovery process after injury. Finally, through GSVA analysis, we found that cluster 0 has significant differences in functions such as cell adhesion and protein signal transduction compared to other groups, which may reflect the special functions of these cells in the recovery process after injury. These findings provide a new perspective for us to understand the cell dynamics after AAO injury.

In conclusion, our study elucidates the unique gene expression signatures of OPC subclusters in the context of AAO injury. These findings contribute to a better understanding of the heterogeneity and functional diversity of OPC in the pathology of AAO and provide potential targets for further investigation and therapeutic interventions.

## 4 Discussion

AAO is a common complication, such as in patients with aortic aneurysms or following abdominal surgery. Previous studies investigating organ functional impairment caused by AAO have predominantly focused on acute effects. In this study, we employed behavioral tests, including the rotarod, Y‐maze, and novel object recognition tasks, to assess the long‐term effects of AAO on brain injury. Our findings indicate that AAO does not adversely affect motor function, as evaluated by the rotarod test. However, it does lead to impairments in spatial memory and object recognition, as assessed by the Y‐maze and novel object recognition tests, respectively. Given that the hippocampus is primarily responsible for these cognitive tasks, we conducted histological analyses, which revealed increased neuronal damage in the hippocampus of AAO mice following ischemia–reperfusion. Notably, compared to direct cerebrovascular ligation, such as bilateral common carotid artery ligation (2VO) [39], AAO resulted in relatively minor hippocampal damage, characterized only by nuclear shrinkage, whereas 2VO led to significant loss of hippocampal neurons. To further elucidate the hippocampal response to ischemia–reperfusion following AAO, we employed single‐cell transcriptome sequencing to analyze the cellular and molecular mechanisms involved.

Our study utilized single‐cell RNA sequencing technology to reveal the heterogeneity of astrocyte subtypes in hippocampal injury induced by AAO. These findings align with existing literature on the diverse roles of astrocytes in brain injury, where astrocytes are known to modulate neuroinflammation and neuroprotection through the release of inflammatory and neurotrophic factors [40]. However, our research further refines these responses within the AAO model, revealing specific alterations in astrocyte subpopulations and their gene expression patterns, providing new perspectives on astrocyte roles in specific brain injuries.

The number of cells in astrocyte subtype Cluster 0 increased significantly following AAO injury, indicating their important role in the injury response. This cluster showed high expression of Gfap and Aldh1l1. Typically, increased Gfap expression correlates with astrocyte activation [37, 41], while Aldh1l1 is associated with metabolic changes [38]. This suggests that astrocytes may be involved in energy metabolism adjustment and inflammation modulation post‐injury. Astrocyte subtype 5 expresses genes related to intercellular communication and signal transduction, such as aquaporin‐4 (Aqp4) and the glutamate/aspartate transporter (Glast). Aqp4 is crucial for maintaining fluid balance and facilitating cell migration. Its role has been extensively studied in the contexts of cerebral edema and inflammation [42]. Meanwhile, Glast regulates glutamate levels, which are essential for neurotransmission and synaptic function. Alterations in Glast expression have been linked to glutamate toxicity in neurodegenerative diseases [43]. Our research enhances these understandings by identifying specific changes in astrocyte subtypes and their gene expression patterns within the AAO model, providing new insights into the roles of astrocytes in specific brain injuries.

Notably, our study highlights significant changes in astrocyte subtype expression patterns associated with positive neurogenesis regulation post‐AAO. This aligns with findings that specific astrocyte subtype activation post‐cerebral ischemia relates to neurogenesis [44]. These astrocytes may promote neuronal survival and regeneration in damaged areas through the secretion of brain‐derived neurotrophic factor (BDNF) and other neurotrophic factors [45, 46]. Therefore, our findings underscore astrocytes' potential importance in repair processes post‐brain injury. Additionally, following AAO, the cellular response in the hippocampal region is not solely driven by injury factors but also initiates repair mechanisms such as neurogenesis to combat the damage. This dual response highlights the complexity of astrocyte involvement in both injury and recovery processes, emphasizing their significant role in maintaining brain health and function.

Our study also reveals distinct mRNA processing patterns in astrocytes that could impact their gene expression and function. These patterns are linked to neuroinflammation and neurodegeneration in Alzheimer's disease [47] and play a crucial role in acute ischemic stroke, influencing neuroinflammatory responses [48]. Our findings indicate that targeting astrocyte mRNA processing could offer therapeutic benefits for neurodegenerative conditions.

Clinically, these insights into astrocyte heterogeneity and function could inform the development of targeted therapies. By modulating specific astrocyte subtypes, it may be possible to enhance neuroprotection, promote neurogenesis, and mitigate neuroinflammation, offering new avenues for treating ischemic brain injuries and potentially other neurodegenerative conditions.

In the central nervous system, OPCs are crucial for maintaining myelin integrity, essential for proper neuronal function and signal transmission. Under pathological conditions, such as ischemic or traumatic brain injury, OPCs become activated and migrate to injury sites, where they can differentiate into myelinating oligodendrocytes, aiding in remyelination and repair [49]. Altered proportions of OPCs may significantly impact the structure and function of the hippocampus, a region pivotal for learning, memory, and spatial navigation [50, 51]. Our study found a significant increase in OPC proportions following AAO injury, likely reflecting the brain's innate response to recruit these cells to facilitate repair and remyelination.

The heterogeneity among OPC subtypes in response to AAO injury provides crucial insights into their roles in neural repair and pathology. OPC0, marked by genes such as myelin basic protein (Mbp), plays a key role in maintaining hippocampal integrity under normal conditions [52] and contributes to neuronal function maintenance through efficient myelination [53]. Post‐AAO, the increased proportion of OPC1, which expresses genes like glial cell line‐derived neurotrophic factor (Gdnf) vital for neuronal survival and repair, suggests its early role in neuroprotection and reparative mechanisms, mitigating ischemic effects [54]. OPC2 mainly expresses pro‐inflammatory cytokines, such as interleukin‐1 beta (Il‐1β) and tumor necrosis factor‐alpha (Tnf‐α), linked to neurodegenerative changes, indicating its involvement in synaptic loss and neuronal dysfunction [55]. The rise in OPC3 post‐injury hints at its role in intermediate repair, involving cellular communication and signal transduction [56]. Significant changes in OPC4 post‐AAO may contribute to long‐term recovery, with gene expressions related to proliferation, differentiation, and remyelination, such as Pdgfrα, NG2, and OLIG1, highlighting its importance in prolonged neural repair and oligodendrocyte lineage maintenance [57].

These findings underscore the dynamic and multifunctional nature of OPCs in the context of AAO injury. The distinct roles of OPC subtypes at various stages of injury suggest potential targets for therapeutic intervention, emphasizing the importance of subtype‐specific modulation to enhance remyelination and synaptogenesis in ischemic and neurodegenerative conditions [31].

In our study, we observed an increase in oxidative phosphorylation in epithelial cells following AAO injury. This pathway is critical for cellular energy metabolism, and its upregulation suggests a compensatory mechanism to meet the heightened energy demands during injury and recovery [58, 59]. Clinically, this finding is significant as it highlights potential targets for therapeutic intervention aimed at modulating energy metabolism to improve cell survival and function.

The enhancement of oxidative phosphorylation may influence the course of AAO injury by affecting epithelial cell function and survival, which are vital for maintaining the integrity of the blood–brain barrier and preventing further neuronal damage [60]. Therapeutically, targeting oxidative phosphorylation could help stabilize cellular energy levels, thereby reducing the risk of cell death and promoting recovery [61].

Furthermore, understanding the role of oxidative phosphorylation in epithelial cells can inform the development of antioxidant therapies [62]. These therapies could mitigate oxidative stress [63], a common consequence of increased oxidative phosphorylation, thereby protecting against further cellular damage and improving clinical outcomes in patients with AAO‐induced injuries.

Although there have been several single‐cell studies related to ischemia–reperfusion injury in existing research [64, 65, 66], however there is no one about to AAO injury. Our study on AAO‐induced hippocampal injury highlights a distinctive aspect of ischemia/reperfusion (I/R) injury, where systemic hypoxia affects the brain due to abdominal aortic occlusion. This contrasts with the localized ischemia seen in the middle cerebral artery occlusion (MCAO) model, as demonstrated by Kai Zheng et al. [67], where there is acute, localized cerebral ischemia leading to robust inflammation and cell death, particularly in neurons and microglia. Another study on cerebral ischemia–reperfusion injury highlights the role of Leucine‐rich alpha‐2 glycoprotein 1 (Lrg1) in modulating various cellular components, including the blood–brain barrier (BBB) and microglia, in a model of focal cerebral ischemia [64]. In contrast, our scRNA‐seq analysis in the AAO model shows upregulation of astrocytes and OPCs with gene expression related to neurogenesis and alternative splicing.

Comparing with the 2VO model, which emphasizes OPC differentiation and protein synthesis [68], our AAO model suggests different cellular adaptation and repair mechanisms, underscoring the variability in cellular responses across ischemic models, and providing insights into diverse mechanisms of brain injury and recovery. This comparison highlights the importance of model selection in ischemic research.

## 5 Conclusions

In conclusion, our scRNA‐seq analysis has significantly advanced the understanding of AAO‐induced hippocampal injury by elucidating the complex cellular dynamics and molecular pathways involved. The identification of specific cell types and gene signatures provides valuable targets for therapeutic strategies. Building on the findings of this study, future research should validate the identified molecular targets using in vivo models and clinical samples. This validation is essential to confirm their relevance to AAO injury and therapeutic potential. Additionally, employing gene editing technologies, such as CRISPR/Cas9, to manipulate the expression of these molecular targets in cell cultures and animal models will be crucial. These studies will provide a mechanistic understanding of how manipulating these targets affects cell function and neural repair in the context of AAO. Lastly, exploring the therapeutic potential of targeting these pathways in preclinical AAO models will be important to assess the efficacy of pharmacological or gene therapy interventions.

## Author Contributions

Changhong Ren designed the entire experiment; Ling Kui and Guoyun Wang analyzed sequencing datasets; Jun Xu and Fang Tong performed the animal experiments and pathological tissue analysis; Xiaojie Wang, Xiaomei Tian and Jianping Ma interpreted the experimental results; Ling Kui, Jun Xu, and Changhong Ren wrote the manuscript; and Xunming Ji, Sijie Li and Fen‐Yong Liu supervised this project.

## Ethics Statement

Experimental animals are raised by the Comments of Animal Experiments and Experimental Animal Welfare Committee of Capital Medical University (approval number: 2021/255). All experimental procedures are strictly in accordance with the International Ethical Guidelines and the National Institutes of Health Guide for the Care and Use of Laboratory Animals.

## Conflicts of Interest

The authors declare no conflicts of interest.

## Supporting information


**Figure S1.** t‐SNE plots visualizing the expression distribution of selected marker gene for neuron (A), excitatory neuron (B) and inhibitory neuron (c).
**Figure S2.** The expression levels of GFAP and NG2 were up‐regulated in AAO mice. A, the graph represents quantitative analysis of GFAP‐positive cell numbers. B, the graph represents quantitative analysis of NG2‐positive cell numbers. Each data represents mean ± SEM, *n* = 3, *p < 0.05, ****p < 0.0001.
**Figure S3.** UpSet plot showing unique or overlapping DEGs derived from the comparison between AAO and Sham samples within each cell type. The black dot represents the DEGs that are shared by more than two cell types. The black bar above each plot represents the number of DEGs for each category.
**Figure S4.** River plot depicting ligand–receptor expression pattern of incoming strength in Sham (A), AAO (B), and outcoming strength in Sham (C), AAO (D) interactions using CellPhoneDB. Size indicates strength, and color indicates the cell type and patterns.
**Figure S5.** A, Diagram represents clustering trees can also be produced directly from Seurat objects of astrocyte, illustrated by nodes of varying colors and sizes. Each color represents clusters under different resolution, with the intensity of the color indicating the count of cells. The size of each node corresponds to number of cells. B, Bar graph displays the correlation of various genes with CytoTRACE. Genes are listed on the *y*‐axis, and their correlation values are represented on the *x*‐axis. Positive correlations are indicated by red bars extending to the right, while negative correlations are represented by blue bars extending to the left.
**Figure S6.** A, Diagram represents clustering trees can also be produced directly from Seurat objects of OPC, illustrated by nodes of varying colors and sizes. Each color represents clusters under different resolution, with the intensity of the color indicating the count of cells. The size of each node corresponds to number of cells. B, Bar graph displays the correlation of various genes with CytoTRACE. Genes are listed on the *y*‐axis, and their correlation values are represented on the *x*‐axis. Positive correlations are indicated by red bars extending to the right, while negative correlations are represented by blue bars extending to the left.

**Table S1.** Differentially expressed gene markers of single‐cell clusters. This table lists the key gene markers identified for different cell clusters in a single‐cell analysis. For each gene, the statistical significance (*p*_val, *p*_val_adj), average log2 fold change (avg_log2FC), and the expression percentages in the two groups (pct.1 and pct.2) are provided. The “cluster” column indicates the associated cell type, while the “gene” column specifies the gene names.

## Data Availability

The data used to support the findings of the current study are available from the corresponding author on reasonable request.
