# Supplementary material for: Single‐Cell Insights Into Cellular Response in Abdominal Aortic Occlusion‐Induced Hippocampal Injury
Source: CNS Neurosci Ther. 2025 Jan 20;31(1):e70154. doi: 10.1111/cns.70154 (PMC11746957; doi:10.1111/cns.70154)
Supplement: Supplementary file 1 — Figure S1. t‐SNE plots visualizing the expression distribution of selected marker gene for neuron (A), excitatory neuron (B) and inhibitory neuron (c). Figure S2. The expression levels of GFAP and NG2 were up‐regulated in AAO mice. A, the graph represents quantitative analysis of GFAP‐positive cell numbers. B, the graph represents quantitative analysis of NG2‐positive cell numbers. Each data represents mean ± SEM, n = 3, *p < 0.05, ****p < 0.0001. Figure S3. UpSet plot showing unique or overlapping DEGs derived from the comparison between AAO and Sham samples within each cell type. The black dot represents the DEGs that are shared by more than two cell types. The black bar above each plot represents the number of DEGs for each category. Figure S4. River plot depicting ligand–receptor expression pattern of incoming strength in Sham (A), AAO (B), and outcoming strength in Sham (C), AAO (D) interactions using CellPhoneDB. Size indicates strength, and color indicates the cell type and patterns. Figure S5. A, Diagram represents clustering trees can also be produced directly from Seurat objects of astrocyte, illustrated by nodes of varying colors and sizes. Each color represents clusters under different resolution, with the intensity of the color indicating the count of cells. The size of each node corresponds to number of cells. B, Bar graph displays the correlation of various genes with CytoTRACE. Genes are listed on the y‐axis, and their correlation values are represented on the x‐axis. Positive correlations are indicated by red bars extending to the right, while negative correlations are represented by blue bars extending to the left. Figure S6. A, Diagram represents clustering trees can also be produced directly from Seurat objects of OPC, illustrated by nodes of varying colors and sizes. Each color represents clusters under different resolution, with the intensity of the color indicating the count of cells. The size of each node corresponds to number of cells. B, Ba [file CNS-31-e70154-s001.docx]

**Supplementary**


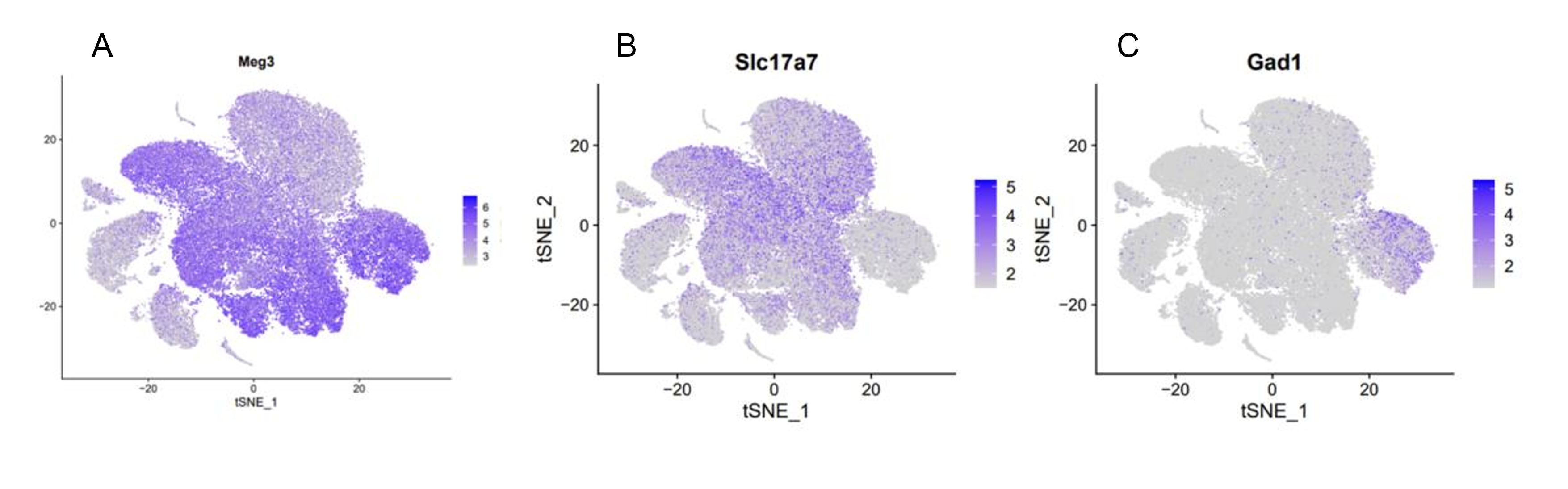
**FIGURE S1**. t-SNE plots visualizing the expression distribution of selected marker gene for neuron (A), excitatory neuron (B**)** and inhibitory neuron (C).


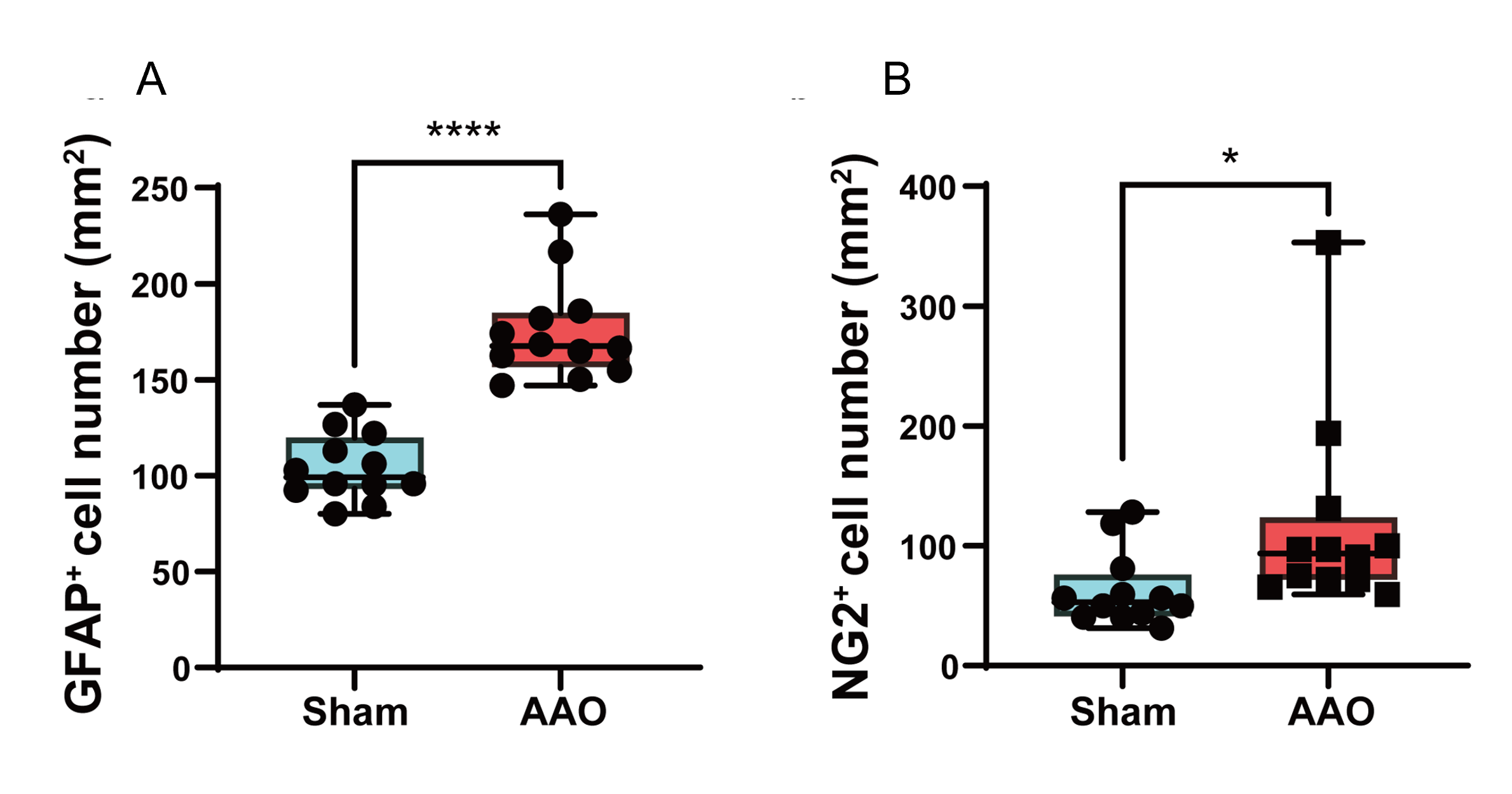


**FIGURE S2**. The expression levels of GFAP and NG2 were up-regulated in AAO mice. **A,** the graph represents quantitative analysis of GFAP-positive cell numbers. B, the graph represents quantitative analysis of NG2-positive cell numbers. Each data represents mean±SEM, n=3, *p<0.05, ****p<0.0001.


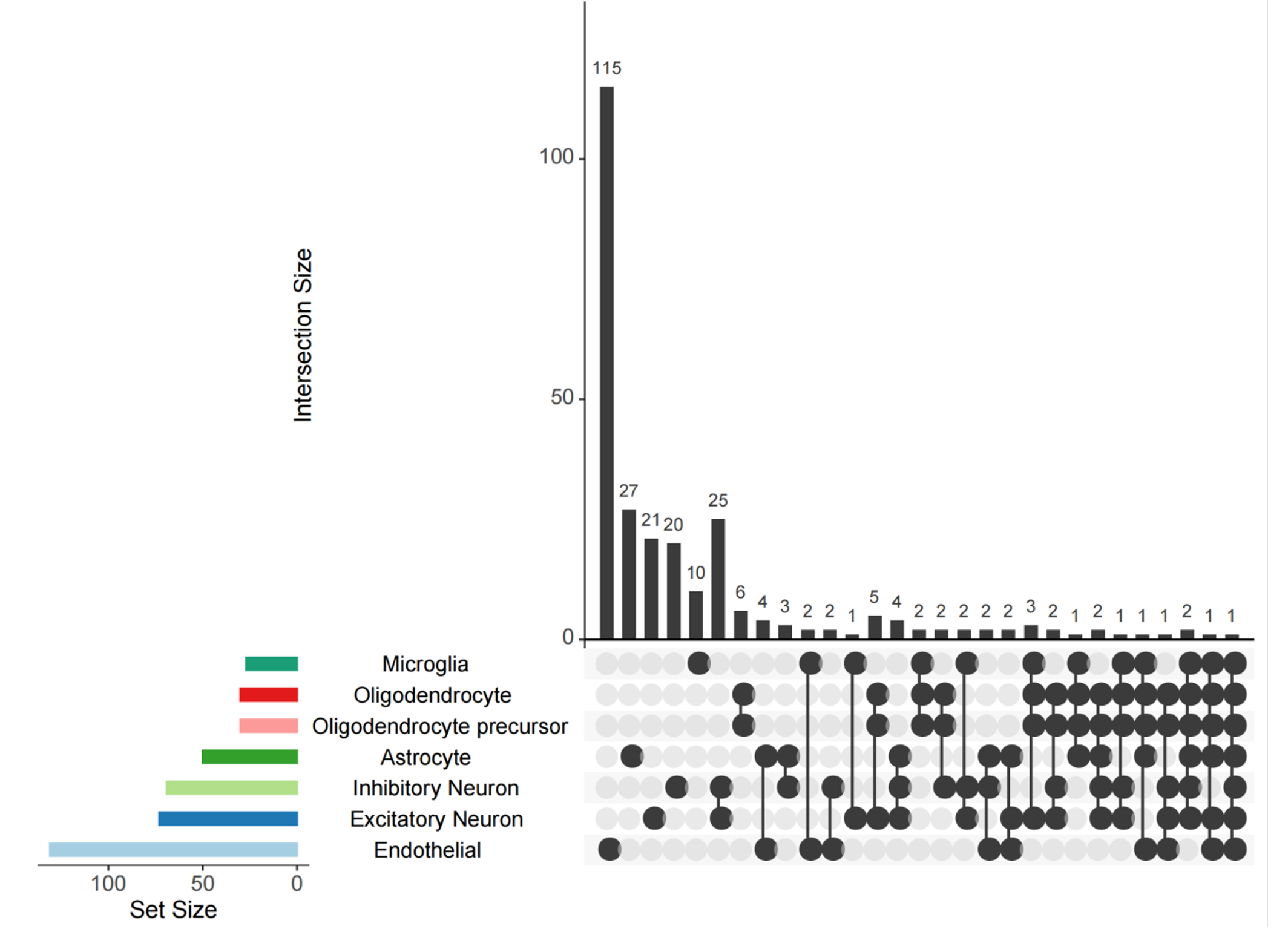


**FIGURE S3**. UpSet plot showing unique or overlapping DEGs derived from the comparison between AAO and Sham samples within each cell type. The black dot represents the DEGs that are shared by more than two cell types. The black bar above each plot represents the number of DEGs for each category.


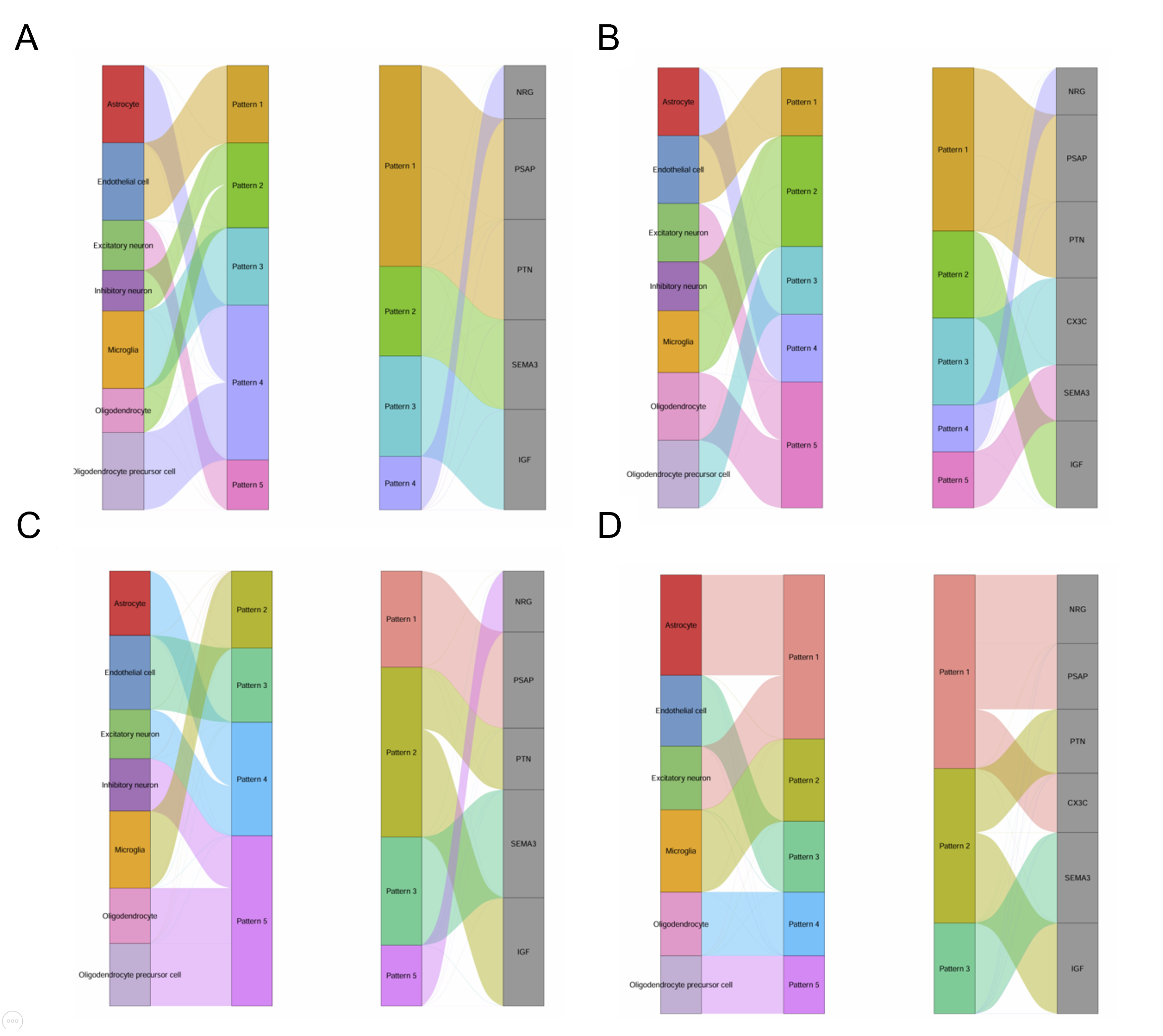


**FIGURE S4**. River plot depicting ligand–receptor expression pattern of incoming strength in Sham (A), AAO (B), and outcoming strength in Sham (C), AAO (D) interactions using CellPhoneDB. Size indicates strength, and color indicates the cell type and patterns.


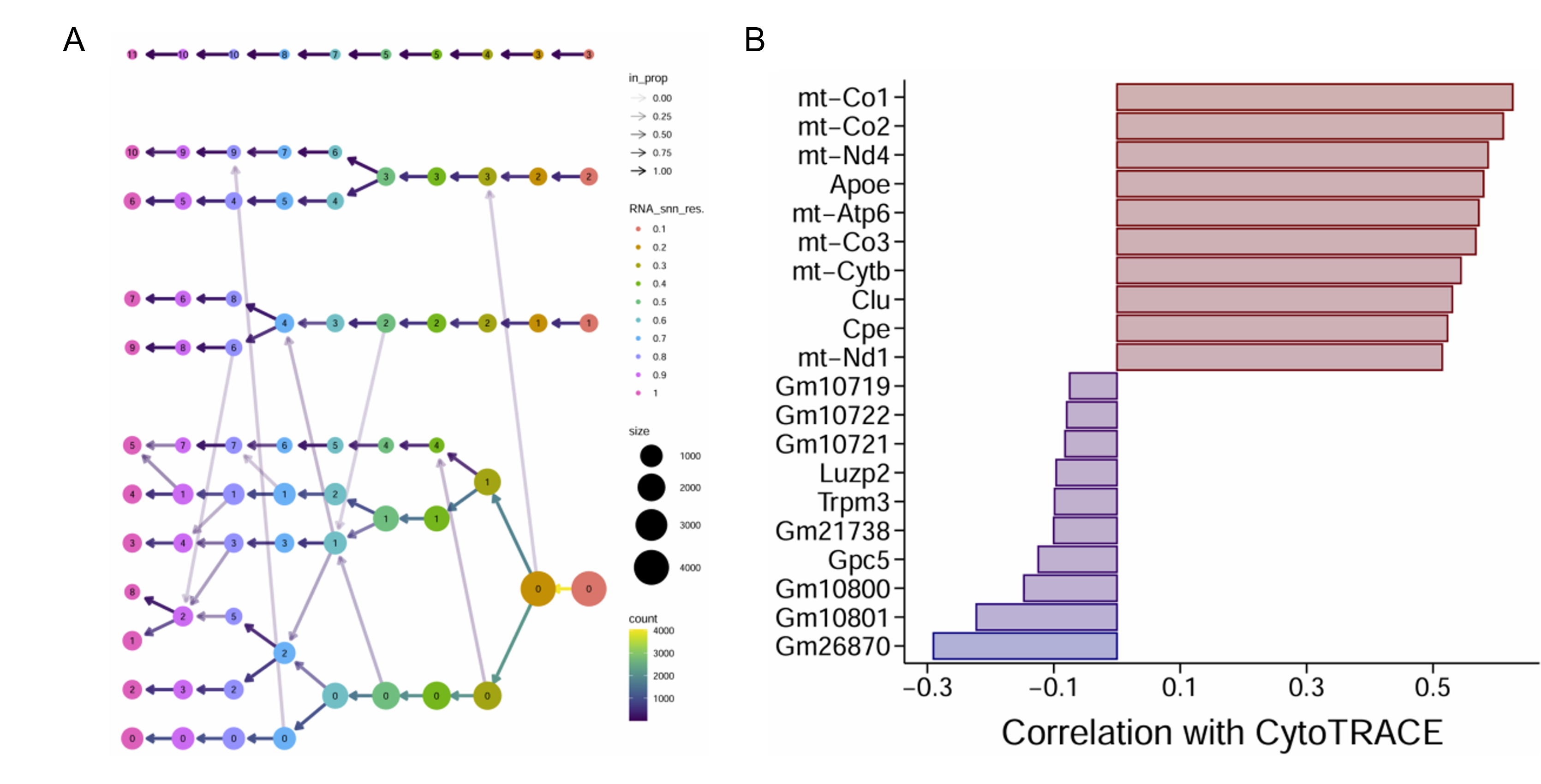


**FIGURE S5**. A, Diagram represents clustering trees can also be produced directly from Seurat objects of astrocyte, illustrated by nodes of varying colors and sizes. Each color represents clusters under different resolution, with the intensity of the color indicating the count of cells. The size of each node corresponds to number of cells. B, Bar graph displays the correlation of various genes with CytoTRACE. Genes are listed on the y-axis, and their correlation values are represented on the x-axis. Positive correlations are indicated by red bars extending to the right, while negative correlations are represented by blue bars extending to the left.


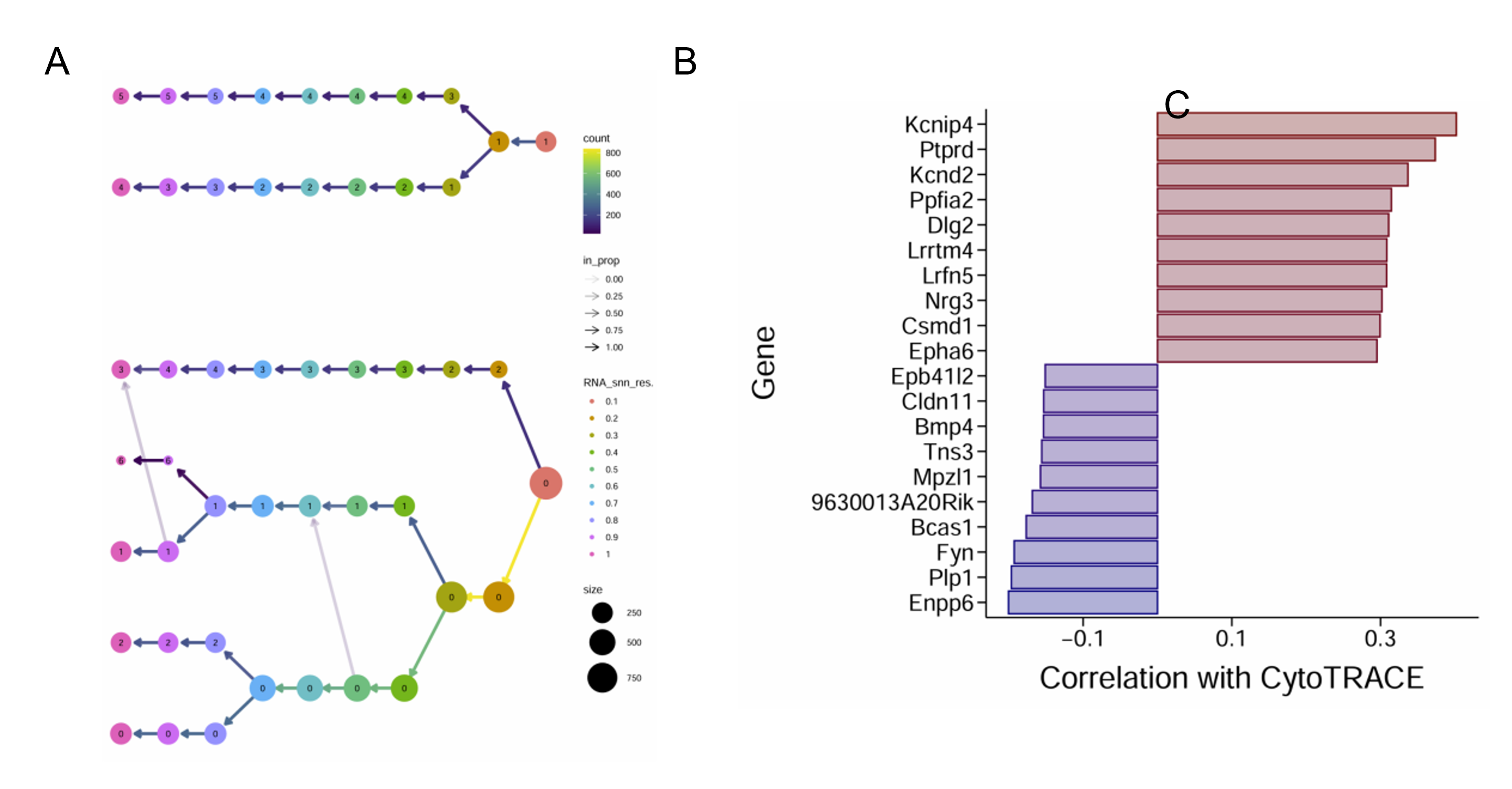


**FIGURE S6**. A, Diagram represents clustering trees can also be produced directly from Seurat objects of OPC, illustrated by nodes of varying colors and sizes. Each color represents clusters under different resolution, with the intensity of the color indicating the count of cells. The size of each node corresponds to number of cells. B, Bar graph displays the correlation of various genes with CytoTRACE. Genes are listed on the y-axis, and their correlation values are represented on the x-axis. Positive correlations are indicated by red bars extending to the right, while negative correlations are represented by blue bars extending to the left.
